# Clients’ experiences of one‐to‐one low‐intensity interventions for common mental health problems: An interpretative phenomenological analysis

**DOI:** 10.1111/papt.12200

**Published:** 2018-10-29

**Authors:** Rebekah Amos, Lydia Morris, Warren Mansell, Dawn Edge

**Affiliations:** ^1^ School of Psychological Sciences University of Manchester UK

**Keywords:** interpretative phenomenological analysis, common mental health disorders, improving access to psychological therapies, low‐intensity interventions, stepped care

## Abstract

**Objectives:**

Common mental health disorders such as depression and anxiety are highly prevalent and carry significant health care and economic burdens. The UK's improving access to psychological therapies (IAPT) initiative was developed as a cost‐effective way of reducing the pernicious effects of these disorders. IAPT interventions, such as guided self‐help, have been subjected to considerable quantitative evaluation. However, there has been minimal investigation into clients’ experiences of the one‐to‐one low‐intensity interventions (LIIs), which form a key component of IAPT service provision. Qualitative exploration could provide rich data regarding experiences of psychological change and factors affecting therapeutic experiences. This will enable informative, client led insights into how low‐intensity therapy can be improved.

**Methods:**

Interpretative phenomenological analysis of eight semi‐structured interviews was used to develop an idiosyncratic understanding of clients’ experiences of one‐to‐one LIIs following entry into a randomized control trial (RCT).

**Results:**

Four superordinate themes were identified from clients’ accounts: goals and expectations of therapy, beneficial aspects of therapy, non‐beneficial aspects of therapy, and the experience of psychological change. A heuristic model of interrelationships between factors is proposed.

**Conclusions:**

Both therapeutic techniques and relationships contribute to beneficial therapeutic experiences. The results reported here can be used to inform practice by harnessing the most beneficial aspects of therapy, such as developing adaptive therapeutic approaches to clients’ clinical needs and facilitating idiosyncratic processes of psychological change. Due to limited qualitative research in this area, further research should be conducted in different service settings to assess differences and similarities in clients’ experiences.

**Practitioner points:**

- Therapists who adapted to clients’ individual needs were perceived as more effective than those who did not.
- Effective therapeutic experiences were exemplified by a personal therapeutic approach, enough time to discuss issues and normalizing client's experiences.
- Clients develop idiosyncratic models of change which should be encouraged by therapists over and above clinical models.

## Background

Common mental health disorders (CMDs) such as depression and anxiety are highly prevalent in the UK and pose significant economic and health care challenges (McManus, Meltzer, Brugha, Bebbington, & Jenkins, 2009). Until recently, CMDs were poorly managed as only 10% of client's accessed appropriate psychological treatment (McManus *et al*., 2009). In response to this lack of provision, the ‘Improving access to psychological therapies (IAPT)’ initiative was developed in 2007 (Clark, 2011).

Improving access to psychological therapies aims to provide nationwide access to empirically validated treatments (Clark, 2011) and follows a ‘stepped care model’ in which the intensity of an intervention matches the severity of one's condition (Bower & Gilbody, 2005). For mild to moderate depression and anxiety, brief low‐intensity interventions (LIIs) are recommended (NICE, 2009). These interventions comprise a selection of computerized cognitive behavioural therapy (CCBT), guided self‐help or one‐to‐one talking therapies (Williams & Martinez, 2008).

Improving access to psychological therapies aims to continually evaluate therapeutic efficacy to ensure treatments lead to effective psychological change (Clark *et al*., 2009; Gyani, Shafran, Layard, & Clark, 2013). Analyses using questionnaire change scores found that within the first year of IAPT, 40.3% of clients achieved ‘reliable recovery’ (Gyani *et al*., 2013). However, recovery rates varied between services (23.9–56.5%) and being offered a higher mean number of sessions was one factor related to greater levels of improvement (Gyani *et al*., 2013). Evidence also indicates that some IAPT practitioners work according to clinical intuition over empirically validated models of practice (Gyani, Shafran, Myles, & Rose, 2014). This suggests variation in IAPT LII's may have differential effects on client outcomes.

Despite the available quantitative evidence of the effectiveness of LIIs (Griffiths & Griffiths, 2015; Gyani *et al*., 2013), quantitative assessments do not capture the nature of change as experienced by clients. Furthermore, the focus on symptomatology reduction as an indication of recovery is not necessarily in line with more client‐centred ideas of recovery, such as living with symptoms (Newbold, Hardy, & Byng, 2013). There is currently a dearth of qualitative research into client experience and psychological change within one‐to‐one LIIs. However, McEvoy, Schauman, Mansell, and Morris (2012) provide important groundwork in establishing the factors involved in psychological change in this context. McEvoy *et al*. (2012) used a mixed methods approach to analyse client experiences of recovery in one‐to‐one LIIs. Principal component analysis indicated that two domains facilitated change – ‘emotional regulation’ and ‘social capital’. Qualitative analysis also revealed the importance of ‘personal goals’, ‘resilience’, and ‘self‐efficacy’. This is largely in line with other change literature (Clarke, Rees, & Hardy, 2004; Higginson & Mansell, 2008; Mansell, 2011) but contributes important context‐specific information about psychological change within one‐to‐one LIIs.

However, there are some limitations with McEvoy *et al*.'s (2012) study. Firstly, the qualitative interviews in the study were brief and data were not analysed by rigorous qualitative methods such as interpretative phenomenological analysis (IPA). Furthermore, this research focused solely on clients who had experienced change. As IAPT LIIs are shown to contribute to recovery in 40.3% of cases (Gyani *et al*., 2013), it is important to also explore the views of those who do not experience change.

### Psychological change: experiences and mechanisms

The experience of psychological change has been shown to vary in terms of how soon clients experience change (Tang & DeRubeis, 1999), the kind of therapy that facilitates change (Beutler, 1999), and the nature of change itself (Newbold, Hardy, & Byng, 2013). There has been a recent focus on understanding these change processes more accurately using rigorous methodologies (Kazdin, 2007) as well as aiming to integrate findings of core similarities into a theoretical model (Andresen, Oades, & Caputi, 2003; Higginson & Mansell, 2008; Higginson, Mansell, & Wood, 2011).

A change in perspective is a common indicator of the occurrence and maintenance of change within qualitative change literature (Higginson & Mansell, 2008). Research suggests that the ability to tackle problems is underpinned by developing new models of approaching problems (Clarke *et al*., 2004; MacDonald, Mead, Bower, Richards, & Lovell, 2007), which may be partially informed by skills and techniques learnt in therapy (Clarke *et al*., 2004). Psychological change has also been shown to be a dynamic process, where change is non‐linear (Tang & DeRubeis, 1999).

An IPA study of clients’ experiences of psychological change indicated that change comprised: a transition from hopelessness to tackling problems; a mixture of sudden and gradual gains; old versus new self; and a change in perspective (Higginson & Mansell, 2008). Within this heterogeneous clinical sample, core factors were found to underlie participants’ experience (Higginson & Mansell, 2008). As such these core processes may be a key area to target (Higginson & Mansell, 2008; Kazdin, 2007; Mansell, 2011).

The relationship between client and therapist has also been shown to positively correlate with client outcome (Orlinsky, Ronnestad, & Willutzki, 2004) such as early symptom reduction (Tang & DeRubeis, 1999). Within IAPT settings, Green, Barkham, Kellett, and Saxon (2014) found that clients were two times more likely to show improvement post‐therapy if they had a more effective practitioner. Despite evidence that therapeutic alliance impacts on clients’ outcomes, this does not suggest that therapeutic alliance is a mechanism of change (Kazdin, 2005).

### Summary and aims

Current research using quantitative paradigms indicates that one‐to‐one LIIs facilitate recovery (Gyani *et al*., 2013). However, there is variance in recovery rates between services (Gyani *et al*., 2013) and the format of therapy, which may affect client outcome (Roth & Pilling, 2008).

A qualitative investigation would enable identification of factors that facilitate or impede psychological change from clients’ perspectives. Qualitative methods yield rich data which can provide novel insights in a given research domain (Smith, Flowers, & Osborn, 1997).

## Method

### Study context

This exploratory, qualitative study was embedded within a 12‐month randomized parallel group trial to establish whether a brief transdiagnostic group, the Take Control Course, was non‐inferior to individual low‐intensity CBT (*N *=* *156). The study gained ethical approval from the National Research Ethics Service (NRES) Committee in 2014 (ref.14/NW/0160).

### Recruitment

All participants, referred to a low‐intensity IAPT service for disorders such as generalized anxiety disorder and depression, provided written informed consent. They comprised a homogeneous sample as all had experienced therapy within a one‐to‐one LII context and at least one therapy session.

Participants were purposively recruited into the study according to the following criteria: sufficient understanding of oral and written English to enable completion of questionnaires, aged 16 or above, appropriate for low‐intensity services (as determined by a 1‐hr clinical assessment with a Psychological Wellbeing Practitioner [PWP]), and having attended at least one session. Exclusion criteria included: experiencing suicidal ideation, psychosis, self‐harming requiring clinical management, substance dependence, organic brain impairment or issues appropriate for high‐intensity referral according to the stepped care model (Bower & Gilbody, 2005).

### Participants

Individual participant characteristics are provided in Table 1. The sample included an equal number of men (*N* = 4) and women (*N* = 4). Seven out of the eight participants were White British and one was Caribbean. Participants’ psychometric data are included to indicate severity of symptoms at the point interviews commenced. Participant's anxiety and depression varied from mild to severe, more severe participants were stepped up to more intensive treatment (See Table 1). We aimed to recruit patients who had attended the minimum (1) to maximum (6+) sessions to explore the effect of session attendance on therapeutic experience. The minimum numbers of sessions attended were 3 and despite aiming to recruit those who attended 1–2 sessions, this was not possible.

**Table 1:** Participant characteristics and indicators of psychological change

Participant	Ethnicity	Gender	PHQ‐9	GAD‐7	Number of sessions received	Stepped up to psychological services?	Qualitative experience of psychological change
Dave^a^	White British	M	**3**	**3**	4	No	Yes
Mike	White British	M	**3**	**3**	4	No	Yes
John	White British	M	**2**	9	6	No	Yes
Julie	White British	F	10	11	6	Yes	Yes
Mark	White British	M	16	15	6	Yes	No
Angeline	White British	F	11	**6**	5	Yes	No
Sarah	Caribbean	F	**3**	**5**	3	No	No
Judith^b^	White British	F	21	14	9	No	No

Notes

Scores indicative of non‐clinical symptomology are highlighted in bold.

a Dave received the other treatment option prior (TCC) to receiving one‐to‐one therapy. His experiences analysed here represent those related to one‐to‐one therapy.

b Judith did not complete 6‐month baseline assessment and was not available to take part in the study at this time point, the data presented are for her 12‐month follow‐up.

Smith (2004) recommends a sample of 5–10 to obtain a rich analysis. The final sample was eight participants, out of 22 who were invited. Given the type of analysis adopted, this sample was considered sufficient to provide a detailed and nuanced exploration of each individual (Smith, 2004).

### Materials

A semi‐structured interview was developed iteratively, based on previous research of expectations towards therapy (Khan, Bower, & Rogers, 2007), psychological change (Higginson & Mansell, 2008; Tang & DeRubeis, 1999), and clinical experience. The following topics were covered:

1. Helpful and/or unhelpful aspects of therapy
2. Associated psychological change
3. Experience of session length.

The interview schedule was piloted opportunistically with volunteers who had accessed one‐to‐one therapy. The interview schedule was initially developed by LM based on the literature and clinical experience. After liaison with the team, prompts were added to support fuller responses. Volunteers suggested that the introductory parts of the interview should be clearer and subsequently this was made more explicit. However, the main topics above remained unchanged. The flexibility of semi‐structured interviews meant researchers could omit or add probing questions to gain unique insights (Smith *et al*., 1997).

#### Measures

Clients’ levels of clinical depression, anxiety, and functioning were measured to demonstrate clinical characteristics shortly before interviews (See Table 1).

##### Patient Health Questionnaire Depression Scale (Kroenke, Spitzer, & Williams, 2001)

A 9‐item scale, with scores ranging from 0 to 27. A score of 10 or above indicates clinical levels of depression. The Patient Health Questionnaire Depression Scale (PHQ‐9) evidences good sensitivity and good internal consistency (Kroenke *et al*., 2001)

##### Generalized Anxiety Disorder Assessment (Spitzer, Kroenke, Williams, & Löwe, 2006)

A 7‐item scale, with scores ranging from 0 to 21. A score of 8 or above indicates clinical levels of generalized anxiety. The Generalized Anxiety Disorder Assessment (GAD‐7) evidences good sensitivity and a specificity for generalized anxiety disorder (Kroenke, Spitzer, Williams, Monahan, & Löwe, 2007).

### Procedure

Clients were recruited from a randomized control trial (RCT; Morris, 2016). RCT participants consented or declined being contacted about this study upon entering the trial. Those who consented were contacted at 6 months post‐baseline assessment via telephone.^1^ Providing participants wished to participate they were offered an appointment.

Eight interviews were conducted, lasting between 15 and 75 min. Interviews were conducted by RA and LM. At the end of interviews, participants were informed that a summary of the study would be available to them online via their services website. Digitally recorded data were transferred from the Dictaphone to a password protected computer, transcribed verbatim and subsequently deleted. Anonymized, transcribed data were imported into NVivo 10 (QSR International, Melbourne, Australia) which supported data management and analysis. The coding framework was developed in a bottom‐up fashion; that is, nodes were used to document all open codes and then further categorized in terms of superordinate/subordinate relationships via parent and child nodes.

### Analysis

Interpretative phenomenological analysis was used to analyse participants’ accounts because it allows exploration of idiosyncratic processes and converging experiences between participants (Smith, 2004). Analysis, conducted by RA, followed the guidelines of Smith (2004). The interviewer read each transcript multiple times to gain understanding of the nature of participants’ accounts. At this stage, potential themes were recorded, as the researcher began to interpret the participant's account. Secondly, initial findings were reviewed by the entire research team and emergent themes organized into a preliminary structure. Thirdly, emergent themes were reviewed to assess possible interrelationships; data were condensed in this phase as a function of focussing on the psychological content of accounts. Fourthly, all cases were compared for convergence and divergence and shared themes were organized. Each case was compared in an iterative manner until final superordinate themes were developed. Pseudonyms have been assigned to each participant to anonymize data (Gough & Lyons, 2016).

To increase the trustworthiness of analyses, several verification strategies were adopted. Firstly, stage two and three of analysis were supervised and reviewed by the research team to ensure data were analysed in accordance with an IPA methodology (Elliott, Fischer, & Rennie, 1999). Secondly, a reflexive dialogue was maintained and each member of the team reflected on their potential biases when interpreting data (Mauthner & Doucet, 2003). RA also recorded upcoming assumptions and monitored researcher bias via memo‐notes (Stiles, 1993). Finally, member checking was conducted to ensure data had been interpreted as participants intended (Tong, Sainsbury, & Craig, 2007). Three key informants (Mark, Mike, and Angeline) took part in member checking, particularly those with unique and varying experiences (Lincoln & Guba, 1985).

## Results

**Figure 1:**
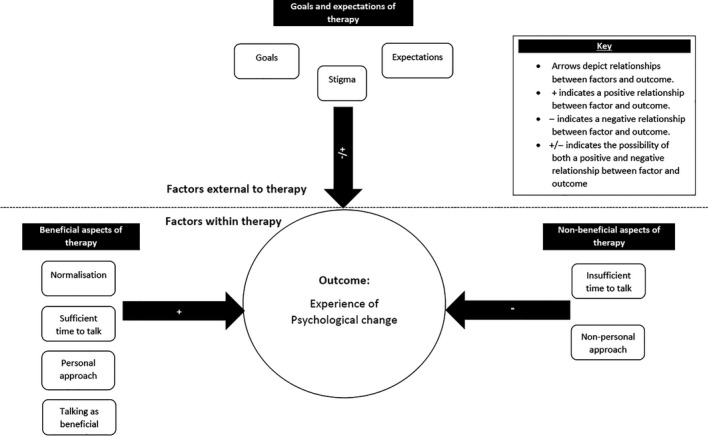
Heuristic model of themes and proposed interrelationships.

Four superordinate themes emerged from participants’ accounts: ‘Goals and expectations of therapy’, ‘Beneficial aspects of therapy’, ‘Non‐beneficial aspects of therapy’, and ‘Experience of psychological change’. As illustrated in Figure 1 and detailed below, there were 14 sub‐ordinate themes. We also provide a detailed case of Julie, which succinctly embodies many of the superordinate themes identified in our analysis (see Box 1).

Box 1 **An in‐depth case overview of Julie, superordinate themes are noted in italics**

1 Julie came to therapy with an inability to cope. She was initially cynical toward the therapeutic process as she didn't want a ‘nicey nicey’ approach (*Goals and expectations*), she knew something was wrong and needed someone to be honest and open rather than overly comforting (*Goals and expectations*).

I didn't want nicey nicey, patronising, you know, I'm, I'm an intelligent woman and I know there is something wrong with me

Julie's distress was typified by a loss of identity. She made it clear that the non‐functional Julie was incongruent with her self‐identity. The psychological impact of this loss of control was profound and unwelcome.

it wasn't me that was sat in that room it's kind of, I always cope.

As such, julie's therapeutic journey was a movement from where she was ‘down there’ toward the Julie that coped (*Goals and expectations*). She acknowledged this was hard and challenging. As such it was important for her to have a therapist who challenged her in a secure and meaningful therapeutic relationship (*Beneficial aspects of therapy*). As she reflected on her recovery, she explained how she had compartmentalised aspects of her personality, that is her ‘angry, emotional, and sensible self’ *(Experience of psychological change)*. In this way, Julie moved closer to the coherent self that mattered – the one that was functional:

I'm a doer, I organise things, I get things done…and I wasn't doing that anymore because the sensible…had gone

Her psychological change was deeply embedded with her sense of identity, whereas other clients focussed more on the use of therapeutic techniques in response to distress.

### Model of interrelationships between themes

A heuristic model of theme interrelationships is provided (Figure 1). Psychological change is placed centrally as an outcome factor, with three pathways. The first pathway, ‘Goals and expectations’, is presented as an external factor. This pathway could be positive or negative depending on the client's previous interactions (or absence thereof) with mental health services. The second pathway, ‘Beneficial aspects of therapy’, includes subthemes which positively influenced psychological change such as sufficient time to talk and a personalized therapeutic approach. The third pathway, ‘Non‐beneficial aspects of therapy’, includes subthemes such as a non‐personal therapeutic approach which negatively impacted client's experience of psychological change. See Figure 1.

### Goals and expectations of therapy

This superordinate theme relates to clients’ expectations for therapy based on prior interactions with services. Goals varied but focussed on psychological improvement. Subordinate themes are as follows: (1) individual goals for therapy, (2) diverse expectations of therapy, and (3) stigma.

#### 1 Individual goals for therapy

Individual goals for therapy varied, but included wanting to reduce emotional distress and increase day‐to‐day functioning:

I wanted to, my words, find my mojo again […] being able to function on a day to day basis without … you know things going wrong and crying

(Julie)

The word ‘Mojo’ underpins Julie's ability to influence something within her environment, which due to its intangible nature was difficult to pinpoint. Here, Julie is aware that the mojo which helped her previously is gone, that is her ability to control her emotions.

Clients also spoke of wanting to become more knowledgeable about the physiological impact of their disorders to make sense of the potential psychological causes:

when I was having my panic attacks from my anxiety, I had never had them before in my life and …. I wanted somebody to help, to explain what was happening to my body

(Mark)

#### 2 Diverse expectations of therapy

Most participants had no concrete expectations of what therapy would entail. Many were unsure how therapy would be structured, with some people expressing initial anxiety and cynicism:

I don't know what I expected it to be like, but I expected myself to be […] nervous, or anxious

(Mike)

I was surprised when I came out of the first session, I actually had hope, and I wasn't expecting that.

(Julie)

However, other participants felt that their previous experience had shaped their interaction with the current therapy. John and Sarah had very positive previous experiences, by comparison the current sessions were somewhat inferior:

the first session, it just felt really strange, because maybe I had gone in with previous expectations […] yeh, where I was gonna get helped and I was gonna get advice

(Sarah)

Sarah explains that she expected to ‘get helped’ and in contrast felt ‘really strange’, due to the incongruent nature of the two therapeutic approaches she had received. The previous therapist had been directive enough to challenge Sarah in areas that needed to be challenged. Whereas, the current therapist provided minimal direction at a time when her overwhelming emotions made it difficult for her to guide herself – ‘I still needed a bit of a steer’.

#### 3 Stigma

Stigma was evident across cases to varying degrees of specificity. Notably, it was male clients who expressed their experiences of social stigma most clearly. There was also an element of self‐stigma across genders. Therefore, stigma here relates to that which occurs socially and internally.

Mark, Dave, and Mike expressed initial feelings of embarrassment when talking about their mental health as they expected others to judge them negatively and as a result would alter the way people perceived them. This affected their willingness to seek psychological treatment:

I just got the point where I said to my mum and dad like “look … you know I'm gonna have to sorta do something about this” […] and my mum and dad were a bit apprehensive about taking me to the doctors at first cus of obviously sorta maybe the stigmas attached

(Dave)

Dave draws clearly here on the stigma from others and his self‐stigma. He had to reach ‘the point’ at which the symptoms had impacted his life to such an extent that he was obliged to discuss these problems. Similarly, Julie points to her experience of mental health issues in a self‐stigmatizing way, below is how she described herself before getting help:

floppy, emotional, pathetic

(Julie)

Mike was also nervous when discussing mental health issues with doctors who were perceived to be practitioners who address tangible and physical problems opposed to psychological issues:

I was nervous going in to tell the doctor … you know what but I wouldn't have been if you know I had my foot (referring here to a physiological and visible medical problem) … but for some reason people are reluctant to … let the doctors know aren't they?

(Mike)

### Beneficial aspects of therapy

This superordinate theme relates to aspects of therapy perceived as beneficial and contributing to positive therapeutic experiences. Subordinate themes are as follows: (1) talking as beneficial, (2) sufficient time for therapy and (3) a personal therapeutic approach, and (4) normalization.

#### 1 Talking as beneficial

Most participants described talking as the most important feature of therapy. The simple act of talking seemed to provide great benefit:

Just the very act of talking seems to work for me ….

(John)

I can go home from her [therapists] sessions, same as I've talked to you, I can go home … and if it's only for a couple of hours and I think, oooh now I've got that off my chest, thank goodness

(Judith)

It was important that participants felt that the therapist was engaged with what they were saying and responding to them accordingly. For Judith, talking gave her temporary release from problems that burdened her. Like John, sharing problems and being listened to was enough to derive a benefit. It was this process of talking and being listened to that was most important:

Even if you were somebody I'd never met before, and you were off the street and you had no skills about what you were doing […] even though you're just listening and paying attention, and asking me little questions … that is brilliant, that is … is, is where I get my help

(‘Mark’)

For Mike and John, it was useful talking to a therapist because, unlike relationships with family members, the therapeutic relationship required minimal self‐censorship, without altering pre‐existing relationships:

If you are talking to a family member or something like that, there is always those preconditions, you've got and you always, whereas in a sense if it's a stranger […] you have the ability to just, say what you're thinking in a non‐judgemental way, and I think, I think that's really useful.

(‘John’)

#### 2 Sufficient time for therapy

For all participants, it was important that they had not been ‘rushed’ (Dave). Not all participants felt they received sufficient time to talk, those who did, felt being able to sufficiently discuss topics within the sessions was an important part of therapy:

There wasn't a point where I ever felt rushed or […] or anything, so me personally I thought yeh. I were given plenty of time … to discuss things

(Dave)

Some participants felt they were not as ‘severe’ as others (Mike) and as such the short number and duration of sessions was satisfactory. These participants also highlighted that the time assigned to therapy suited them personally, but may vary between others:

They were ok for me, for what I, for my needs, I don't think I was you know, the most, the most er… err… serious, but you know, the most desperate…er?

(Mike)

#### 3 Personal therapeutic approach

Therapy was perceived as beneficial when therapists seemed actively attentive to clients and their circumstances. It was important that the interaction was ‘personal’ (Dave, Julie, Mike, Mark) and that therapists seemed genuinely interested in clients beyond the responsibilities of their job:

There was no disruption, there was no looking at a computer, there was no reading notes […] it was eye to eye, so he was paying attention to me […]it was almost as like, it wasn't his job

(Julie)

Julie gives an image of a very attentive therapist, not distracted by extraneous tasks associated with their role. ‘It was eye to eye’ gives a sense of physical connectedness between the two with a level of intimacy that allowed her to feel truly listened to. The therapist held a genuine interest in her care beyond his professional responsibilities.

John echoes the personal relationship Julie describes. John draws attention to the almost instantaneous nature of engagement:

It's probably just down to them (the therapist), and their attitude….some people you kind of, you kind of shine to and some people you don't shine to as much, that's, the, the thing.

(John)

The use of the word ‘shine’ invokes an image of warmth and light. The ability to talk to someone does not seem dependent on their skills, but their quality as imbued in the therapeutic interaction.

#### 4 Normalization

Normalization involved therapists educating clients about mental health generally. This allowed them to feel that others may have problems in common and improved their ability to talk about mental health issues:

First of all just talking to someone I think, initially, is…erm, helpful, you know, feeling… knowing that … it's not, you know… err unusual what you're doing

(Mike)

Dave ‘She (the therapist) made me feel as though I wasn't on my own like, I wasn't…’

Interviewer Right

Dave ‘you know, there are a lot more people that are in my situation sort of thing …’

It was important that clients felt that others experienced similar problems that their problems were not ‘unusual’. This process of normalization counteracted participants initial stigmas, resulting in them becoming less embarrassed about their condition.

### Non‐beneficial aspects of therapy

This superordinate theme relates to aspects of therapy perceived as non‐beneficial and contributing to negative therapeutic experiences. Subordinate themes are as follows: (1) insufficient time for therapy and (2) non‐personal therapeutic approach. These aspects of therapy were often linked to a lack of experience of psychological change, as well as a mismatch between the therapy and the clients’ initial goals and expectations (see Figure 1).

#### 1 Insufficient time for therapy

Some participants felt that sessions were too brief and impeded their ability to explore issues through talk. For John, the restraint on time within sessions interjected his ‘journey’ halting his ability to explore his problems:

John ‘as you start talking, you start thinking and you can reflect, so you can go, you kind of go on a journey …without the journey quite abruptly…kind of halted, and then waiting for the next time’

Interviewer ‘so it was almost kind of halted?’

John ‘I guess in the sense it felt a little bit like that, whereas with an hour, you definitely feel after an hour you've talked a lot, and you've explored a lot’.

Mark and Angeline felt that questionnaires took up time which could have been used to discuss how they had been since the last session. They were unable to explain to the therapist what was important from their perspective:

I thought, that the amount of paperwork that I had to fill in, the circling the one to sevens, the one to fives…I found that it took up too much time for myself personally, and it didn't leave enough time, just… just for her to say, “well how have you been?”

(Mark)

#### 2 Non‐personal therapeutic approach

Some participants felt that they did not connect with their therapist. These participants felt therapists were working according to set protocols opposed to a genuine desire to help:

it did feel a bit, bit more as part of a process this time round, as opposed to, wanting to help.

(John)

Not everybody fits a particular, like, model approach really, and you can adapt them, but also it's just being careful to listen to the …client really

(Sarah)

Perceiving therapists as working according to a process was interpreted as lacking adaptation to clients as individuals. This leads to Sarah and John feeling as though they were not listened to.

### Experience of psychological change

This superordinate theme relates to client's experience of psychological change as a result of one‐to‐one LII. Psychological change was a deeply personal process, related to the presence of beneficial/non‐beneficial therapeutic factors occurring within and outside of therapy (See Figure 1).

Beneficial factors outside of therapy such as stigma and a mismatch between therapeutic expectations were associated with a lack of psychological change. Beneficial factors within therapy which positively impacted change included sufficient time to talk and a personal therapeutic approach. Whereas, a limited time to talk and a non‐personal approach were associated with a lack of psychological change. Therefore, beneficial therapeutic factors better facilitated psychological change, whereas non‐beneficial factors impeded it.

Subordinate themes are as follows: (1) change as gradual, (2) change as continuous, (3) gaining perspective, and (4) idiosyncratic approaches to dealing with problems.

#### 1 Change as gradual

Most participants felt that change was a gradual and incremental process, where each session lead to an improvement:

It's like a weight being lifted of your shoulders, it's like a little bit lifted, and it was little bits at a time, each and every time

(Julie)

When talking about his increased ability to deal with new challenges and to reduce his ‘over‐thinking’, Dave explains that the feeling of change did not ‘click’. He was able to acknowledge his change in retrospect but he was not focally aware of a point when change had happened:

It wasn't like a eureka moment, there wasn't a moment where it just like clicked

(Dave)

Participants felt change was something to be worked on, which took effort to maintain as there was no ‘instant fix’ (Mike).

#### 2 Change as continuous

Change was described as a journey that starts once therapy begins (John, Julie, Dave), but does not necessarily end when therapy ends. John, who had a previous life‐changing therapeutic experience and had relapsed, conceptualized his current therapy as a ‘refresher’. He was not starting anew:

I guess it wasn't as grandiose, as the previous time. […]as in, you know, just a bit of fill up on the motorway services thing, so it wasn't really, it didn't really have a major impact it's just necessary to remember the journey you're on

(John)

This metaphor of journey is extended by Julie below:

You know it's kind of like I'm on the road now, I don't want to come off it

(Julie)

‘The road’ Julie is describing can be interpreted as the road to wellbeing. Coming off the path can be thought of some instability on this journey such as distress or relapse. Julie has gained the ability to manage her emotions and thoughts, but recognizes that continued effort is needed:

Julie I don't want that… ‘oh god here we go something else’ person to come back

Interviewer do you think that persons gone?

Julie no not entirely

#### 3 Gaining perspective

After therapy, participants were able to put aside thoughts that bothered them before. Clients gained an ability to see problems differently and not ‘over‐think’ (Dave) or get ‘angry’ (Julie) unnecessarily. Specifically, after therapy, they were able to ‘push away’ (John) thoughts that were not so important:

There is the place where I am now, they (the thoughts) don't dog my mind anymore, they don't play on my mind, because obviously I've just sort of learned to let go…and focus on what's important

(Dave)

By developing a new ability to push some of the less important thoughts away, problems were moved to the periphery of one's mind.

#### 4 Idiosyncratic approaches to dealing with problems

The way clients approached problems after change was different for each person. For example, ‘Julie’ conceptualized herself as having ‘sensible’, ‘emotional’, and ‘angry’ selves, where the ‘emotional self’ had quietened down and the ‘sensible self’ now dominated:

I said ‘no, we're not gonna do that, if it is worst case scenario, deal with it at the time, but for now we don't know what it is, so for now we are going to be calm’ […]and it's like there is somebody else in my head talking to me […]and that is my current coping mechanism

(Julie)

Here, ‘Julie’ is subconsciously dividing her personalities based on their emotional quality, allowing her to use each perspective from each personality in turn.

Other participants utilized techniques such as mindfulness but adapted it to their individual needs by varying the context and frequency of use.

## Discussion

This study provides insight into clients’ experiences of one‐to‐one LIIs and associated psychological change. Change was experienced as a gradual and continuous process, marked by a new change of perspective. As a heuristic model emerged from the data, it became apparent that this process of change was central to all clients’ experiences. Subsequently negative and positive factors within and outside of therapy affected this experience of change.

The gradual and continuous nature of change described by clients’ contrasts with previous literature that suggests significant periods of change are sudden in nature (Tang & DeRubeis, 1999). However, within a LII context research suggests clients experience change as a combination of sudden and gradual moments (McEvoy *et al*., 2012). Therefore, clients within LII contexts can experience change in incremental stages upon which progress is accumulated within each session.

The finding that psychological change was marked by changes in perspective is in line with previous recovery literature (Clarke *et al*., 2004; Higginson & Mansell, 2008). Literature indicates that acquiring the ability to change perspective to rationalize problems from several angles features in participants with variable mental health difficulties (Higginson & Mansell, 2008). Therefore, this ability seems important in managing problems more effectively across disorders and can be used as a marker of psychological change.

Participants who experienced change developed idiosyncratic approaches to deal with their problems. This varied from adapting mindfulness techniques, to re‐conceptualizing mental self‐images. Other literature also suggests that clients adapt learned techniques according to their individual needs (Khan *et al*., 2007; MacDonald *et al*., 2007). This suggests that clients implement the most salient elements of therapeutic techniques. Positive therapeutic factors such as providing clients with enough time to talk whilst adapting to their individual needs facilitated the change process. A criticism of LII treatments is that PWPs who deliver interventions are trained to apply therapeutic approaches determined by disorder‐specific criteria (Binnie, 2015). As such, practitioners should ideally develop an adaptive approach to treatment. However, PWPs may vary in confidence when deviating from a pre‐specified treatment approach (Binnie, 2015).

We also found that goals and expectations which were external to therapy influenced clients’ interaction with services. As the model (see Figure 1) indicates, stigma negatively impacted clients’ psychological change. Normalizing patient experiences counteracted the negative impact of stigma and indirectly reduced its negative impact on psychological change thereafter. The pernicious effects of stigma need to be reduced to allow clients exploration of their mental health issues (Khan *et al*., 2007). Our research suggests that stigma can be reduced during the early stages of accessing primary care services via a General Practitioner (GP). It may be particularly important for GPs to destigmatize mental health for male patients as they expressed the impact of stigma more clearly than their female counterparts in our study. Men generally have more stigmatizing attitudes of themselves and hold more stigmatizing views of male depression in contrast to women (Oliffe *et al*., 2016). Future research could investigate the differential impact of stigma on men and women's experiences of LII.

We also found that clients who subjectively experienced sessions as too short were less likely to experience overall therapy as effective. A report by Mind (2010) revealed that only 50% of patients accessing psychological interventions felt that the sessions were long enough. This contrasts literature that proposes brief sessions are reliably beneficial for this population (NICE, 2009). Gyani *et al*. (2013) also found that clients were more likely to recover if they had been offered a higher than average number of sessions. Some participants viewed completing questionnaires as too time‐consuming. Although psychometric measures provide important clinical information to therapists (Clark, 2011; Gyani *et al*., 2013), this might not be well received by clients. One solution might be to educate clients as to how and why questionnaires are used as this has led to improved experiences of therapy delivery elsewhere (Khan *et al*., 2007; Mitchell & Gordon, 2007).

Our findings also indicated that clients’ experiences of the therapist were an important factor within therapy. Clients who felt a personal connection with their therapist reported positive experiences. Our model also indicates the importance of this factor in supporting effective psychological change within therapy. This is similar to findings in ‘therapeutic alliance’ literature that a collaborative connection with the therapist influences therapeutic outcome (Orlinsky *et al*., 2004). Therefore, within LII settings it is important not to overlook the importance of the engagement process.

### Strengths and limitations

This research has provided, to our knowledge, the first qualitative study to explore factors facilitating or inhibiting positive therapeutic experiences and associated psychological change in one‐to‐one LII's. By using an IPA methodology to interpret complex psychological processes, this study revealed unique insights into clients’ experiences. The idiographic focus of an IPA methodology allowed us analyse participant experiences grounded by their own perceptions. We were able to identify factors that both impeded and facilitated positive therapeutic experiences. Practitioners can use our heuristic model to assess the impact of both negative and positive factors within their own practice.

Another strength of the study was the implementation of validity measures. Meetings with the research team facilitated a nuanced analysis and a reflexive dialogue regarding potential biases (Stiles, 1993). Secondly, memo‐notes allowed RA to review theoretical thoughts at several stages of analysis (Mauthner & Doucet, 2003). Via member checking participants gave feedback on the study analysis and whether they felt it reflected their experiences. Participants reached a consensus that the researcher's analysis was congruent with their experiences (Tong *et al*., 2007).

However, there are several limitations. The first limitation is sample size. A sample of 5–10 participants is acceptable within IPA, due to the in‐depth analysis involved (Smith, 2004). However, a larger sample size may have facilitated a more nuanced analysis. Secondly, participants included here had been through several stages of an RCT prior to taking part. This sample may not represent the views of people who did not attend sessions. Furthermore, those who did not take part may have had particularly negative therapeutic experiences. However, including participants who had experienced a mid‐number (3) to maximum number (6+) of sessions allowed identification of divergence between clients. A further limitation regarding the sample is that one participant (Dave) had received treatment from both arms of the RCT. However, this participant's most recent therapeutic experience was of the one‐to‐one LII, and throughout the interview featured as the most effective form of therapy he had received.

Another limitation is that the sample was mainly White British. The effect of ethnicity on clients’ perceived efficacy of treatment could not therefore be explored. However, within the north‐west which the service is embedded, the population is 92% White British (Young & Sly, 2010). This sample therefore reflects the majority of the population accessing this service.

### Conclusion

This research provides a heuristic model of psychological change in an LII therapeutic context (see Figure 1). This model may be used in a one‐to‐one LII context to capitalize the most beneficial therapeutic factors which are associated with better psychological outcomes. This research suggests that whilst one‐to‐one LII's support psychological change, flexibility is required when a therapist chooses a therapeutic approach and when allocating time per client. However, such flexibility may be difficult to implement given the current structure of IAPT services nationwide. Given the dearth of qualitative literature in this area, it is recommended that further qualitative studies are conducted in diverse LII settings.

## Note

1 One participant ‘Judith’ did not complete 6‐month baseline assessment and was not available to take part in the study at this time point, and the data presented are for her 12‐month follow‐up.
